# Dynamic Comparison of Segmentary Scapulohumeral Rhythm Between Athletes With and Without Impingement Syndrome

**DOI:** 10.5812/iranjradiol.14821

**Published:** 2014-05-15

**Authors:** Cyrus Taghizadeh Delkhoush, Nader Maroufi, Ismail Ebrahimi Takamjani, Farzam Farahmand, Ali Shakourirad, Hamid Haghani

**Affiliations:** 1 Department of Physical Therapy, Faculty of Rehabilitation Sciences, Iran University of Medical Sciences, Tehran, Iran; 2 School of Mechanical Engineering, Sharif University of Technology, Tehran, Iran; 3 Advanced Diagnostic and Interventional Radiology Research Center (ADIR), Tehran University of Medical Sciences, Tehran, Iran; 4 Faculty of Health Management and Information Science, Iran University of Medical Sciences, Tehran, Iran

**Keywords:** Acromioclavicular Joint, Biomechanical Phenomena, Shoulder Impingement Syndrome

## Abstract

**Background:**

Patients who have shoulder pain usually have compensatory or contributory deviation of shoulder motion during arm elevation. In the traditional scapulohumeral rhythm, the share of the acromioclavicular (AC) and the sternoclavicular (SC) joint movements and also the role of AC internal rotation angle are unknown.

**Objectives:**

The main purpose of this study was to measure and compare the segmentary scapulohumeral rhythm (SSHR) during scapular arm elevation at a steady rotational speed in athletes with and without impingement syndrome.

**Patients and Methods:**

Using a speedometer, the maximum speed of arm elevation was measured in 21 men in each of the involved and uninvolved groups. Using fluoroscopy on the dominant side, SSHR during scapular arm elevation at a rotational speed equal to 1/30 of maximum speed was compared between the two groups. The ratio of glenohumeral (GH) elevation angle to AC rotation angle in the scapular plane was considered as SSHR.

**Results:**

The maximum speed of arm elevation between the two groups was significantly different (P < 0.001). The rhythm of the involved group significantly exceeded the rhythm of the uninvolved group in a part of the first quarter range of the arm elevation. SSHR during arm elevation in the uninvolved group did not change significantly (P = 0.845); however, it decreased significantly in the involved group (P = 0.024).

**Conclusions:**

Speed differences between the two groups were probably due to the pain in some ranges of arm elevation. SSHR in the involved group probably changed in order to compensate downward rotation of the scapula in the resting position. Study of the AC upward rotation range can be misleading; therefore, the study of scapulohumeral rhythm is recommended.

## 1. Background

Shoulder disorder is important both individually and socially, because it may lead to health care and economic costs due to impairmentof psychosomatic health and sickness absences (1, 2). It seems that reduction of the subcoracoacromial space during arm elevation due to alteration of the shoulder motion pattern induces shoulder impingement and creates inadequate space for the rotator cuff tendons and other subacromial structures (3, 4).

Patients who have shoulder pain usually have compensatory or contributory deviation of shoulder motion during arm elevation (5). Scapulothoracic (ST) motions are acquired from combined motions of the acromioclavicular (AC) and the sternoclavicular (SC) joints (4-7). It is presumed that the mentioned joints adjust the rhythm for the best corresponding position of the scapula to provide optimal glenohumeral (GH) joint performance, and change their ranges to modify the humerothoracic range (8). It might be supposed that reduction of the subacromial space due to superior humeral head migration during arm elevation (9) could be compensated by further scapular upward rotation angles through the osteokinematic pattern of the AC and the SC joints or one of them because the lateral border of the acromion process moves far from the humeral head.

ST upward-downward rotation has been measured in dynamic condition using the fluoroscopic method (10-14), invasive (5, 15), and noninvasive (16-21) techniques of electromagnetic tracking method. In the static condition, it has been measured using fluoroscopic (22), radiographic (23), electromagnetic tracking (6, 24, 25), and magnetic resonance imaging (26, 27) methods.

AC upward-downward rotation has only been measured by invasive (5) and noninvasive (7) techniques of electromagnetic tracking method. Studying rotational motion of the scapula in static condition would be certainly different from dynamic and active conditions regarding kinematic and kinetic perspectives.

Validity of the electromagnetic tracking method in the measurement of scapular rotation needs more investigations (7, 28) due to considerable skin movement over the scapula (5, 7, 15, 16, 20), skin sweating (20), and measurement errors more than recorded ranges (7) in noninvasive techniques and also pain, numbness, and range of motion limitation in noninvasive techniques(5).

In the previous studies of rotational motion of the scapula, the speed of arm elevation either was not controlled (12-14, 21) as confounding variable or it was controlled poorly using discrete auditory feedbacks (7, 20), self selected speed, or learned speed (11, 18, 19, 24). Control of the speed during arm elevation and among individuals is required due to the alteration of active and passive tissue tensions and neuromuscular control pattern in various speeds (29, 30). Reliable and valid confirmation or rejection of the motion pattern alteration in the segments of the shoulder girdle are clinically encountered challenges (4, 5, 13, 28); whereas, identifying involved joint or joints will lead us to better planning and goal oriented treatment instead of planning for all joints in the shoulder girdle. Rotational motion of ST has been compared between individuals with and without impingement syndrome in static (27) and dynamic (19) conditions using the electromagnetic tracking device. Correcting abnormal patterns of ST rotation instead of purposeful treatment in the AC or SC joints are aimed in current therapeutic protocols (28). Rotational motion of the AC joint has only been studied in healthy individuals up to 120 degrees of arm elevation (5, 7) and to our knowledge, no comparison has been made between individuals with and without impingement syndrome using the fluoroscopic method.

## 2. Objectives

The main purpose of this study was to measure and compare segmentaryscapulohumeral rhythm (SSHR) during scapular arm elevation at a steady rotational speed in athletes with and without impingement syndrome. Another purpose was the calculation of intra-rater reliability for the analysis.

## 3. Patients and Methods

### 3.1. Patients and Controls

Twenty one male volunteers from wrestling clubs in Tehran participated in each group of healthy and shoulder impingement syndrome with an average age of 24.67 ± 3.68 and 22.05 ± 3.83 years, respectively. Rotational motion was studied on the dominant side in both groups (12). Each athlete read and signed an informed consent approved by the Medical Ethics Board of Iran University of Medical Sciences before enrollment. Individuals were enrolled by an orthopedic surgeon and a physical therapist based on established inclusion and exclusion criteria by previous studies in each group (9, 31, 32). Inclusion and exclusion criteria are mentioned in Table 1. In the involved group, an onset of more than 21 days and pain severity less than 4,based on the visual analog scale (VAS) was considered as inclusion criteria because protective motor reorganization would probably be resolved following acute pain alleviation (4, 29, 30). The average elapsed time from onset was 10.16 ± 15.68 months in the involved group.

**Table 1.** Inclusion and Exclusion Criteria

**Inclusion Criteria in the Involved Group and Exclusion Criteria in the Uninvolved Group**
Pain onset more than 21 days without any treatment during this period
Pain in rest or movement less than 4 based on the visual analog scale (VAS)
Pain in active arm elevation
Pain worsening in one of the static resistance tests; abduction, external and internal rotations
Pain worsening in Neer test or Hawkins Kennedy impingement test
**Exclusion Criteria in Both Groups**
Musculoskeletal involvement history such as dislocation, subluxation and fracture
Surgery or treatment history in the cervical, shoulder or chest region
History of systemic, neuropathy or myopathy disease
Positive compression or distraction test and radiculitis or radiculopathy
Abnormal shoulder range of motion in each cardinal plane
Positive sulcus or load and shift test and instability of the shoulder joint
Positive posterior internal impingement test and internal impingement of the shoulder joint
Observation of degenerative changes in hard or soft tissues in recorded images

### 3.2. Instrumentation

Movement pattern was recorded using fluoroscopy (OP-114 model, SHIMADZU Corporation, Japan) with a C shaped arm. The C arm was placed in the horizontal plane and its distance to the floor was 110 cm. The images were collected at a rate of 15 images per second. Roughly, the dose of radiation was 80 and 38 mgy. cm^2^ for the involved and uninvolved groups, respectively. Image resolution was 300 PPI. Data were calculated using constructed program in MATLAB and Excel software (9, 31, 32).

Speedometer system (Faculty of Mechanical Engineering, Sharif University of Technology, Tehran, Iran) was made of a semicircle skeleton. Seventy one lamps were mounted in the inner wall of a C shaped skeleton from zero to 180^o^ at equal distances. The radius of the C shaped skeleton was 90 cm and its central distance to the floor was 110 cm. The center of the semicircle skeleton could be determined in the space using a glassy ruler installable on the main skeleton. A pair of arms in 15^o^ and a pair of arms in 165^o^ toward the center of the semicircle skeleton were mounted on the semicircle skeleton in order to measure the speed. Each pair of arms was equipped with a laser source and a laser sensor. Reliability of the speedometer device was determined in a pilot study and it was excellent.

The maximum output of the laser in the pointer was less than 50 mW, and its wave length was 532±10 nm.

### 3.3. Speed Controlling Method

A speedometer device was used to measure the maximum speed of arm elevation and to control the steady speed during motion. Athletes sat on a chair in a way that the trunk support was in contact with the opposite side of the trunk below the armpit. The seat was fixed and could not rotate. The speedometer device was placed in the scapular plane (30 degrees counter clock wise rotation from the frontal plane on the right side and 30 degrees clock wise rotation from the frontal plane on the left side). The seat height was adjusted in a way that the lateral angle of the acromion process coincided on the semicircle skeleton center. Each athlete raised his arm at the maximum speed between the two mentioned pairs of arms. The mean speed of arm elevation was automatically calculated based on laser cutting moments in 15^o^ and 165^o^ (speed = 150/elapsed time for each person). In order to control the steady speed during motion and speed normalization, and to have a similar speed to other studies, the rotational speed of the light was adjusted based on 1/30 of the maximum arm elevation speed for each athlete. After determination of the rotational speed, the lamps in the inner wall of the speedometer turned on and off at a specific sequence so that every moment the light was composed of two lamps that induced imagination of a goal movement equal to the ordered speed. Then, each athlete followed the light motion using a laser pointer in his hand with his elbow extended.

Goal directed arm elevation was practiced between 5 to 10 times in the determined speed before recording in order to refine it. Motion was recorded after 5 minutes rest.

### 3.4. Imaging Method

**Figure 1.**
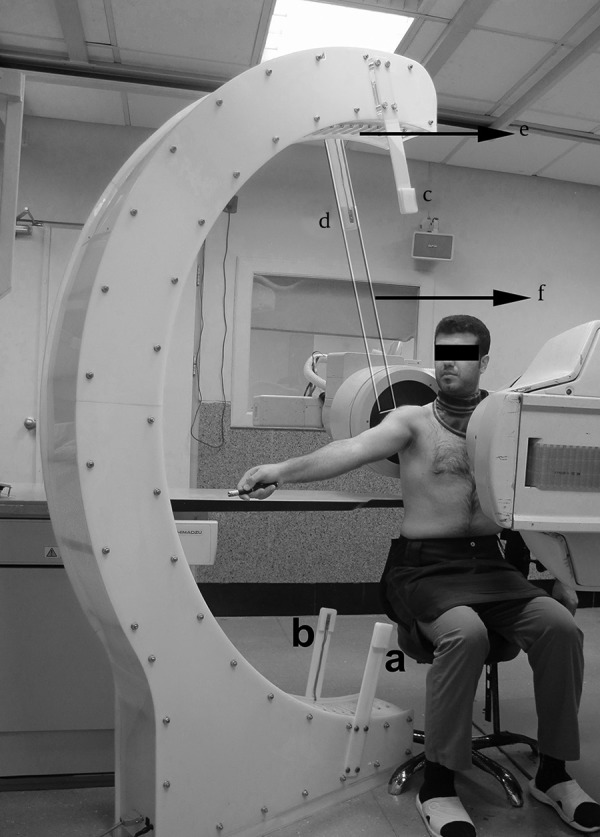
Experimental set up during fluoroscopic recording. a)The arm in 15^o^ contains the laser source, b) The other arm in 15^o^ contains the laser sensor, c) The arm in 165^o^ contains the laser source, d) The other arm in 165^o^contains the laser sensor, e) Lamp series in the inner wall, f) Glassy ruler

Each athlete wore a lead cover and sat on the seat so that trunk support was in contact with the opposite side of the trunk below the armpit (Figure 1). The seat was fixed and could not rotate. The posterior wall of the chest was positioned 2 cm away from the image intensifier (9, 32). For pixel calibration, in the intended shoulder a 4.4 mm diameter lead bullet was attached on the skin of the lateral angle of the acromion process. The speedometer device was placed in the scapular plane. The seat height was adjusted so that the lateral angle of the acromion process coincided on the speedometer center. One image from the initial and one from the final ranges of the arm elevation were taken to ensure that all segments moved in the field of the view. The seat height and position for each athlete were adjusted again, if needed. Goal directed arm elevation at the defined speed was repeated three times, and only the second time was recorded. During recording, no motion was imposed to the athletes to ensure the natural motion pattern. Images were collected in the scapular plane similar to the previous studies (9, 31, 32). In this purpose, the C arm was placed in the horizontal plane and rotated 30 degrees counter clock wise around the vertical axis from the sagittal plane on the right side and rotated 30 degrees clock wise around the vertical axis from the sagittal plane on the left side to radiate waves perpendicular to the scapula bone. No degenerative changes were observed in hard or soft tissues in the recorded images.

### 3.5. Image Analysis

Bony landmarks were manually determined using the manual digitization method in each image (9, 31-33). In this way, the superior and inferior edges of the glenoid fossa were connected to each other in order to draw the glenoid line. The conoid tubercle and the inferior edge of the external end of the clavicle were connected to each other in order to draw the clavicle line.

To calculate the AC upward-downward rotation angle, 90° was subtracted from the angle between the glenoid line and the clavicle line. Positive and negative values were considered as scapular upward and downward rotations, respectively.

**Figure 2.**
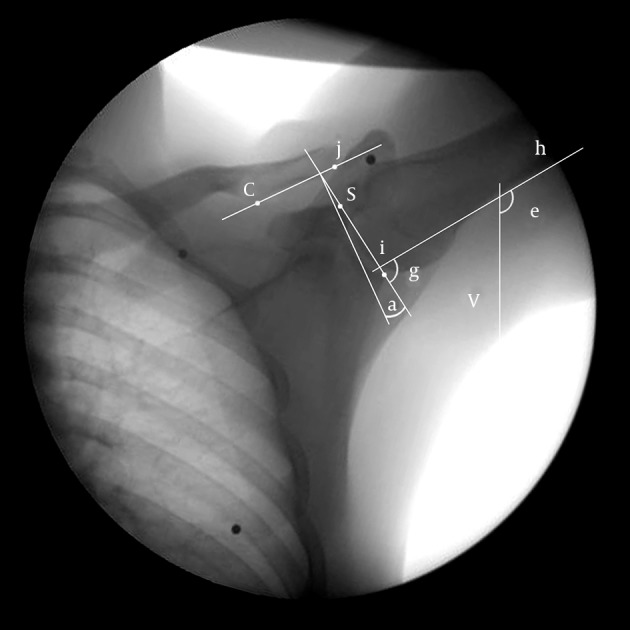
Glenoid line is drawn between superior (s) and inferior (i) ridges, clavicular line is drawn between conoid tubercle (c) and the AC joint (j), the angle (g) between the humeral line (h) and the glenoid line (si) represents the GH elevation angle, the angle (e) between the humeral line (h) and the vertical line (v) represents the arm elevation angle, and the angle (a) between the glenoid and clavicular lines minus 90 degrees indicates the AC rotation angle.

The arm elevation angle was measured between a line drawn on the medial side of the humeral shaft (humerus line) and the perpendicular line on the image (9, 31, 32). The GH angle was measured between the humerus line and the glenoid line (Figure 2). Reverse SSHR was calculated by dividing the AC rotation angle to the GH elevation angle multiplied by hundred. In some instances, the AC rotation angle was zero; therefore, instead of the rhythm, the reverse rhythm was used in calculations. Calculations in 5 degree intervals in the arm elevation range were compared between the groups.

### 3.6. Reliability Analysis

At first, point placement in all recorded images from the first 5 participants was practiced by a rater (9, 31). To calculate point placement variability, frames in the beginning, 25%, 50%, 75%, and the end of the total motion range (5 images) from an athlete were selected and propagated 5 times. Then, the images were randomly arranged so that no image was placed after another. The mean of coefficient of variation (CV) in the measurement of the AC rotation and the GH elevation variables was 2.6% and 0.8% in two measurements and it was reduced to 1% and 0.6% in five measurements, respectively. It was decided to apply the average of two measurements in analyses due to negligible differences between the two and five measurements.To calculate the intra-rater point placement and measurement reliability, frames in the beginning, 25%, 50%, 75% and the end of the total motion range (5 images) from five uninvolved and five involved athletes (a total of 50 images) were selected and measured twice in a 2-week interval. Intra-class correlation coefficient (ICC) and standard errors of the measurement (SEM) were calculated for the AC rotation and the GH elevation variables (Table 2).

**Table 2.** Intra-Rater Reliability Results ^a^

Arm Elevation (%ROM)	ICC		SEM(^o^)	
	AC rotation	GH elevation	AC rotation	GH elevation
**0**	0.99	0.99	0.29	0.32
**25**	0.99	0.99	0.27	0.40
**50**	1	0.99	0.00	0.36
**75**	0.99	0.99	0.28	0.41
**100**	0.99	0.99	0.28	0.32

^a^ Abbreviations: AC, Acromioclavicular; GH, Glenohumeral; ICC, Intra-class Correlation Coefficient; SEM, Standard Error of Measurement; ROM, Range of motion

### 3.7. Data Analysis

Data were analyzed by SPSS for Windows software, version 10 (SPSS Inc., Chicago, Illinois, USA). Kolmogorov-Smirnov test was used to analyze the normality of the distribution of the variables. To compare the mean of rhythm between the two groups, two-tailed independent t-test was used in the normal distribution; otherwise, the Mann-Whitney test was used. Arm elevation variable was considered as an independent variable and reverse SSHR was considered as a dependent variable. A P value lower than 0.05 was considered significant. Data were presented as a mean±SD (95% confidence interval).

**Figure 3.**
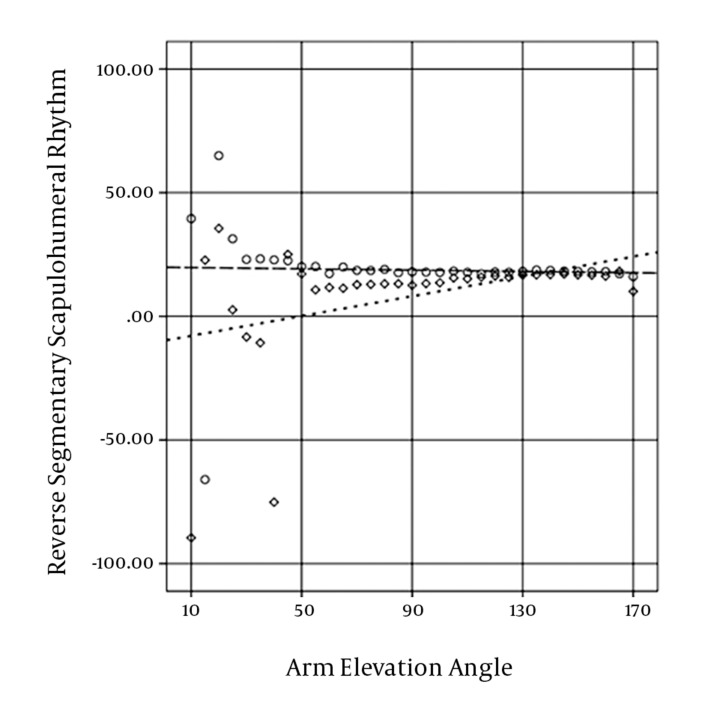
Reverse SSHR during arm elevation; circle represents uninvolved athletes, and rhombus represents involved athletes

The correlation coefficient was used to determine the relationship between arm elevation and rhythm (Figure 3), the AC rotation, and the GH elevation in both groups.

## 4. Results

The mean maximum arm elevation speed in the uninvolved and involved groups was 969.76 ± 103.58 (924.53 to 1014.99) and 457.31 ± 59.31 (431.41-483.21) degrees per second, respectively. The maximum speed of arm elevation between the two groups was significantly different (P < 0.001).

Initial angle of the GH elevation was 3.33 ± 5.17 (1.08-5.58) and 7.92 ± 7.03 (4.85-10.99) degrees in the uninvolved and involved group, respectively; and final angle was 111.5 ± 6.01 (108.87-114.13) and 106.77 ± 5.86 (104.21-109.33) degrees in the uninvolved and involved group, respectively. Differences between the two groups in initial (P = 0.216) and final (P = 0.339) angles of the GH elevation was not significant.

Initial angle of the AC rotation was 5.85 ± 9.82 (1.56-10.14) and -5.55 ± 7.32 (-8.75 to -2.32) degrees in the uninvolved and involved group, respectively; and final angle was 18.07 ± 7.89 (14.61-21.53) and 10.20 ± 19.35 (1.75-18.65) degrees in the uninvolved and involved group, respectively. The difference between the two groups in the initial angle was significant (P = 0.021), but it was not significant in the final angle (P = 0.333).

Except for 20^o^, 35^o^ and 40^o^ angles in the involved group, data had a normal distribution in all arm elevation angles. In addition to the mentioned angles, the nonparametric test was used to compare the mean between the two groups in the initial and final 10^o^ of arm elevation due to the inadequate sample size.

The mean of reverse SSHR between the two groups was significantly different in 35^o^ in the probability value of 0.05 (P = 0.024) and in the probability value of 0.10; it was significantly different in 30^o^ (P = 0.074), and 40^o^ (P = 0.054).

Reverse SSHR during arm elevation in the uninvolved group did not change significantly (P = 0.845), but it increased significantly in the involved group (P = 0.024).

The scapula in the AC joint in the uninvolved and the involved group rotated upward significantly (both Ps < 0.001). Humerus in the GH joint in the uninvolved and the involved groups elevated significantly (both Ps < 0.001).

## 5. Discussion

SSHR during arm elevation in the uninvolved group did not change significantly, but in the involved group, it decreased significantly. The rhythm of the involved group significantly exceeded the rhythm of the uninvolved group in the first quarter range of arm elevation (from 10^o^ to 50^o^ of arm elevation).

Reliability was excellent and consistent with previous studies (22, 23). Reliability of the AC rotation variable in the middle range of arm elevation exceeded initial and final ranges of arm elevation probably due to anatomical structure overlap in the initial and final ranges of arm elevation. SEM was consistent with the finding of Massimini and colleagues (34).

At present, the study of GH elevation on ST rotation ratio is more popular (10-13, 28). In the mentioned studies, the scapula and the clavicle were considered integrate in the calculation of scapulohumeral rhythm; whereas, ST upward rotation depends on the AC upward rotation, the SC elevation, and the posterior axial rotation. Reported differences between the mentioned studies could be a result of disregarding the AC internal rotation angle as a confounding variable.

**Figure 4.**
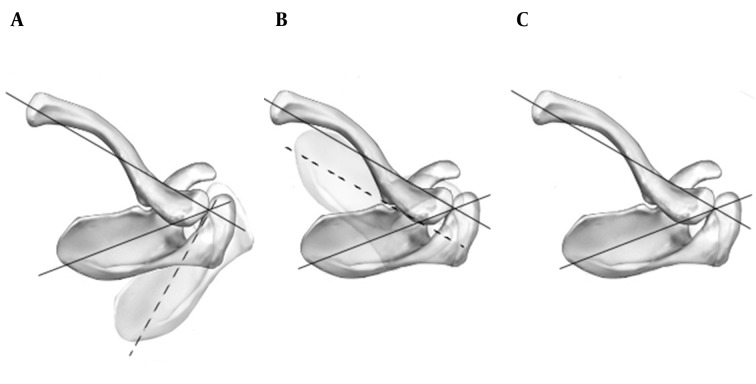
A) AC internal rotation angle was assumed 90o. B) AC internal rotation angle was assumed zero. C) Normal AC internal rotation angle in the horizontal plane

If the scapular plane was assumed perpendicular to the clavicular long axis (Figure 4 A), elevation of the clavicle would be directly coupled with scapular anterior tilting as well as clavicular posterior axial rotation and scapular upward rotation; and if the scapular plane was assumed parallel with the clavicular long axis (Figure 4 B), clavicular elevation and scapular upward rotation would be directly coupled as well as the clavicular posterior axial rotation and the scapular posterior tilting (7, 28).

It was demonstrated that the AC internal rotation angle in healthy individuals was approximately equal to 75% of the assumed 90^o^ (Figure 4 C); therefore, a combination of 75% of the assumed 90^o^ and 25% of the assumed 0^o^ should theoretically occur with clavicular rotation (7).

Thus, the effect of clavicular elevation and posterior axial rotation in scapular upward rotation depending on the AC internal rotation should be different in various individuals.

Teece and colleagues showed that in the AC joint, the scapula rotated upwardly 14.6º during arm elevation from 10º to 90º, and the scapula rotated upwardly 7º in the resting position (7). Ludewig and colleagues demonstrated that the scapula in the AC joint rotated upwardly 11º during arm elevation from 20º to 120º, and the scapula rotated upwardly 5º in the resting position (5). Based on extrapolation from their graphical results, it could be inferred that SSHR of healthy individuals increased during arm elevation.

In the present study, the scapula in the uninvolved group rotated upwardly 5.85º in the resting position, which was consistent with the two mentioned studies. The scapula in the AC joint rotated upwardly 4.86º and 8.18º during arm elevation up to 90º and 120º, respectively; which were inconsistent with the mentioned parallel studies. SSHR in 90º and 120º of arm elevation was 5.57 and 5.59, respectively, which was compatible with the last rhythm of mentioned parallel studies, respectively. However, the rhythm of the uninvolved group in the present study did not change significantly (P = 0.845); whereas, the rhythm increased in the two mentioned studies.We believe that rhythm differences between the present and the two mentioned studies probably originated from measuring methods and the differences could not be explained completely by the degree of freedom (3).

Reduction of scapular upward rotation induces the reduction of the subacromial space and it leads to the development or progression of shoulder impingement (3, 4, 7). In the present study, in the resting position, the scapula showed an upward rotation of 5.85º in the uninvolved group and a downward rotation of 5.55º in the involved group. Less AC upward rotation angles in the first quarter range of arm elevation due to downward rotation of the scapula in the resting position in the involved group was probably an induced reduction of the subacromial space in the first quarter range of the arm elevation.

Despite what is common; prediction of the subacromial space based on the upward rotation range of the scapula is misleading. The upward rotation angle of the scapuladepends on the resting position of the scapula and the upward rotation range of the scapular. In the first quarter range of the arm elevation in the present study, the scapula in the AC joint rotated upwardly 0.3º and 7.39º in the uninvolved and involved group, respectively. Although the upward rotation range of the scapula in the involved group was more than the uninvolved group, the upward rotation angle of the scapula was 1.84º in the involved group and 6.15º in the uninvolved groupdue to the resting position of the scapula.

In order to correct the subacromial space and to reduce pain (3), probably the rhythm in the involved group showed a compensatory decrease in the present study. A rhythm up to 25º of arm elevation varied in the present study, and also sample sizes in the groups below 20 degrees of arm elevation was disproportionate due to various starting angles of arm elevation. A rhythm below 25º of arm elevation between the two groups was not significantly different probably due to the mentioned reasons.The average of the maximum speed in the involved group decreased significantly, which was probably due to the pain in some ranges of arm elevation (3, 4). In healthy individuals, the average arm elevation speed in the parallel studies was 33.33 (5) and 26.67 (7) degrees per second, while it was 32.33 degrees per second in the current study. Various speeds may lead to different motion patterns (4). Therefore, the results must be compared according to the arm elevation speed. In this regard, the arm elevation speed in healthy athletes of this study was consistent with the study conducted by Ludewig and colleagues (5).

Fayad and colleagues showed that rotational motion of the scapula in healthy individuals was not different between fast and slow arm elevation (18). The maximum speed of arm elevation differs considerably between the current (969º/s) and previous (90º/s) studies. In the previous study, the arm was probably elevated with feasible maximum and minimum speeds of a preferred movement pattern.

In the two dimensional study of the movement pattern, anatomical structures overlap in some ranges of the movement and little geometric transformation due to out of the plane motion are accounted for the limitations of the fluoroscopy method. On the other hand, this method has an acceptable validity (33-35) and reliability (22, 33-35). Movement pattern can be studied dynamically and functionally (13). Fluoroscopy exposes less radiation than conventional methods without a reduction in the diagnostic accuracy (22, 32, 36).

In conclusion, SSHR during arm elevation in the uninvolved group did not change significantly, but decreased significantly in the involved group. The rhythm of the involved group significantly exceeded the rhythm of the uninvolved group in the first quarter range of arm elevation.
